# Immunomodulatory activity of extracts from five edible basidiomycetes mushrooms in Wistar albino rats

**DOI:** 10.1038/s41598-022-16349-2

**Published:** 2022-07-20

**Authors:** Shaza M. Elhusseiny, Taghrid S. El-Mahdy, Nooran S. Elleboudy, Mohamed M. S. Farag, Khaled M. Aboshanab, Mahmoud A. Yassien

**Affiliations:** 1 Department of Microbiology and Immunology, Faculty of Pharmacy, Ahram Canadian University (ACU), 4th Industrial Area, 6th of October City, Cairo, 2566 Egypt; 2 Department of Microbiology and Immunology, Faculty of Pharmacy, Helwan University, Cairo, 11795 Egypt; 3 Department of Microbiology and Immunology, Faculty of Pharmacy, Ain Shams University, Organization of African Unity Street, Cairo, 11566 Egypt; 4 Botany and Microbiology Department, Faculty of Science, Al-Azhar University, Cairo, 11884 Egypt; 5 Armed Forces College of Medicine (AFCM), Cairo, Egypt

**Keywords:** Drug discovery, Immunology

## Abstract

Mushrooms are nutritious foods that are widely cultivated all over the world. They are rich in a range of compounds linked to improving functions of the immune system including carotenoids, alkaloids, lectins, enzymes, folates, fats, organic acids, minerals, polysaccharides, phenolics, proteins, tocopherols, terpenoids, and volatile compounds. In this study we investigated, the immunomodulatory activity in rats of the aqueous extracts of five of the most common edible mushrooms belonging to Family Basidiomycota-white-rot fungi including, *Lentinula edodes, Agaricus bisporus*, *Pleurotus ostreatus*, *Pleurotus columbinus,* and *Pleurotus sajor-caju*. Male Wistar albino rats were assigned to thirteen groups and Immunosuppression was induced by oral administration of dexamethasone (0.1 mg/kg), followed by oral administration of the mushroom extracts at low (200 mg/kg) and high (400 mg/kg) doses. A positive control group received the immune stimulant *Echinacea* extract Immulant® at (30 mg/kg), while the negative control group received only saline. From each animal, in each group, blood samples were collected after 15 days for complete blood counts and for measurement of immunologic parameters, including lysozyme activity, nitric oxide (NO) production and serum cytokines including tumor necrosis factor-alpha (TNF-α), interferon-gamma (IFN-γ) and interleukin 1 beta (IL-1β) levels. Results have shown that white blood cells (WBCs) and lymphocytic counts were significantly boosted by high doses of each of the five mushroom extracts (207–289% increase for WBC and 153–175% for lymphocytes) with a significant increase in lysozyme activity (110–136% increase), NO concentration (159–232% increase) and cytokines as compared to the negative control group. Histopathological examination of the rats' spleen and thymus tissues has shown marked lymphocytic proliferation that was more obvious at the higher doses. In conclusion, our results showed that the five edible mushroom extracts revealed significant immunostimulatory effects preclinically particularly, at the higher doses (400 mg/kg) which can be considered the effective dose.

## Introduction

Malnutrition and infectious diseases are still a concern, particularly in the developing world, because they weaken the immune defenses^1^. Allopathic drugs are commonly applied to boost the immune system^2^. Nevertheless, these treatments are prohibitively expensive, difficult to obtain, and frequently associated with unpleasant drug reactions^3^. Consequently, the vast majority of people particularly in the developing countries, need complementary remedies such as algae and fungi as biological response regulators (BRMs) and immunoadjuvants^4^.

Mushrooms have been widely used for nutritive and remedial characteristics^5^. White rot *basidiomycetes* fungi including *Agaricus bisporus* (*White Button mushroom*), *Pleurotus columbinus* (Oyster Mushroom), *Lentinula edodes* (shiitake mushroom), *Pleurotus ostreatus* (oyster mushroom), and *Pleurotus sajor-caju* (Grey oyster mushroom) have received much attention due to their medicinal properties, which include immune system modulation, hypoglycemic, antithrombotic activity, antihypertensive, anti-inflammatory, antimicrobial, and antitumor properties as well as the ability to lower blood cholesterol levels^5^. White rot fungi are rich in fibers, proteins, vitamin D, B vitamins, potassium, selenium and antioxidants^6^. They have evidenced an impressive pattern of health-promoting actions including anticancer, antihyperglycemic, antihypertensive, immunostimulant, hepatoprotective, neuroprotective, antifungal, antibacterial, antiviral and prebiotic activities^7,8^.

*Basidiomycetes* mushrooms have proven to be immune system enhancers, to strengthen the immune response in animals and humans against parasitic, bacterial and viral infections, inflammation, cancer, and other diseases^9^. Bioactive components having potent effects on the immune system have been extracted from mushrooms and studied broadly, including polysaccharides, glycopeptides, β-d-glucan, protein complexes, terpenoids, and proteoglycans^10–12^. Life-threatening infectious diseases have multiple immune escape tactics, thus limiting the establishment of an effective immune response and resulting in symptomatic infections^13^. Bioactive compounds in mushrooms could activate the innate and adaptive immune responses in pre-clinical studies, and react as bio-response modifiers^14^. Accordingly, the aim of the present study was to evaluate the in vivo immunomodulatory activities of five commonly used edible basidiomycetes mushrooms including, *Pleurotus ostreatus*, *Pleurotus columbinus*, *Pleurotus sajor-caju*, *Lentinula edodes* and *Agaricus bisporus* in an animal model of Wistar albino rats.

## Materials and methods

### Chemicals

#### Chemicals and reagents

Ethanol for extract preservation was supplied from El-Gomhorya Company, Cairo, Egypt; Dexamethasone was obtained from the commercial syrup by Arab drug company ADCO, Cairo, Egypt). *Echinacea* extract, *Immulant®* was commercially available syrup from Mepaco Medifood Pharmaceutical Co., Cairo, Egypt). All solvents and chemicals were of the highest commercially available purity.

#### Kits

Enzyme-linked immunosorbent assay (ELISA) kits for Tumor necrosis factor-α (TNF-α), Interferon gamma (IFN-γ), Interleukines 1 beta (IL-1β) and Nitric oxide (NO) were from Dynatech Laboratories Inc., Billinghurst, UK.

### Mushrooms

Fresh mushroom fruiting bodies of *P. ostreatus, P. columbinus, P. sajor-caju, L. edodes* and *A. bisporus* mushrooms were collected during the spring season from Al-Orman botanical reservoir, Cairo, Egypt. We have previously identified the mushrooms through sequencing of the internal transcribed spacer (ITS) by Sanger sequencer (Applied Biosystems, Foster City, CA, USA) and deposited the sequences in the GenBank® under accession codes MK603976*,* MZ642245, MZ642259, MN622787 and MZ642282, respectively^15,16^.

### Preparation of aqueous mushroom extracts

Mushroom fruiting bodies, 500 g of each, were air dried in daylight for 2 successive says, powdered, macerated for three successive days in distilled water at room temperature (approximately 25 °C), and then filtered till exhaustion. After extraction, the yield was kept in 80% ethanol for preservation. Ethanol was evaporated at 45 °C, then each isolate was freeze-dried to give approximately 20 g of dry residue.

### Animals

Seventy-eight male Wistar albino rats aged 10 weeks and weighing 150–200 g each were used in these experiments. The animals were obtained from the animal house facility of Faculty of Pharmacy, Ain Shams University Cairo, Egypt. They were housed in an air-conditioned room at 22 ± 3 °C, 55 ± 5% humidity and in standard metal cages with alternating light and dark cycles changed at 12 h intervals and supplemented with standard laboratory diet and water. All animal procedures were conducted in agreement with the Guide for Care and Use of Laboratory Animals and received an approval from the research ethical committee of Faculty of Pharmacy, Ain Shams University, Egypt (Protocol approval number: ACUC-FP-ASU-RHDIRB2020110301-Nr. 38). This study was carried out in accordance with the ethical standards laid down in compliance with relevant guidelines the revised Animals (Scientific Procedures) Act 1986 in the UK and Directive 2010/63/EU in Europe). This study was carried out in accordance with ARRIVE guidelines (https://arriveguidelines.org).

### Experimental design

Rats were randomly assigned to thirteen groups; each group consisted of 6 animals. The animals received treatment for thirty days as follows; Group 1 served as a control group, where rats received only normal saline for the 30 days. In groups 2–13, immunosuppression was induced by administration of dexamethasone (0.1 mg/kg; p. o. by stomach tube) for 15 days. For group 2 dexamethasone administration was followed by normal saline for another 15 days, and for group 3 (positive control) *Echinacea* extract at (30 mg/kg; p.o. by stomach tube) for 15 days. In groups 4–13, dexamethasone was followed by the five mushroom extracts for another 15 days. Groups 4, 6, 8, 10, and 12 received low dose (200 mg/kg; p. o. by stomach tube) and groups 5, 7, 9, 11, and 13 received high dose (400 mg/kg; p. o. by stomach tube) of *P. ostreatus*, *P. columbinus*, *P. sajor-caju*, *L.edodes* and *A. bisporus*, respectively. The doses were selected as previously reported by^17^.

From each animal in each group, blood samples were collected after 15 days from the retro-orbital plexus after lidocaine (4%) local anesthesia^18^. Samples were collected using capillary tubes into Ethylenediaminetetraacetic acid (EDTA) and non-EDTA sterile tubes. The portions of blood in EDTA tubes were tested for complete blood cell counts and differentiation using a Beckman automated coulter A-T Pierce hematology analyzer (Beckman Coulter, Inc., Fullerton, CA, USA). The portions of blood in non-EDTA tubes were left for 30 min at room temperature, and then centrifuged (Laborezentrifugen, 2k15, Sigma, Germany) for 10 min at 3000×*g* at for serum collection. The collected serum was used for measurement of immune parameters including: lysozyme activity, NO production and cytokine (TNF-α, IFN-γ and IL-1β) levels. At the end of the experiment, rats of each group were euthanized by cervical dislocation. Spleen and thymus of 2–3 rats from each group were carefully removed and preserved in 10% formalin for histopathological examination.

### Immune biomarkers assays

#### Assay of lysozyme activity

Non-specific and specific immune assays were detected by serum lysozyme activity using the method of Song et al.^19^*.* Detection of lysozyme concentration in blood serum was done when lysozyme diffuse through the agarose gel containing a suspension of *Micrococcus lysodeikticus* ATCC No. 4698*.* Lysoplates were prepared by dissolving 1% agarose in 0.067 M PBS at pH 6.3 and heated at 100 °C till complete dissolution. The agarose was cooled to 60–70 °C, then a uniform suspension of *Micrococcus lysodeikticus* in 5 mL saline was added to 1 L of agarose and mixed well. Plates were poured at thickness of 4 mm and left to cool. Wells, 2 mm in diameter 4 × 4 rows 15 mm apart, were cut in the agarose. Working lysozyme standards were prepared freshly by diluting 3 mL of stock lysozyme solution to 10 mL with 8.5 g/L sodium chloride. The wells were filled with a volume of 25 µL of blood serum samples as quickly as possible. Each filled plate contains the 5 working lysozyme standards as well as the samples to be assayed. The plates were covered tightly and incubated at room temperature on a level surface for 12–18 h. At the end of the incubation period, the diameter of the clearance zone around each well was measured to the nearest 0.1 mm with an enlarge viewer (Kalesttad Laboratories Inc, Austin, Texas, USA). The serum lysozyme activity was measured and expressed as µg/mL.

#### Determination of NO concentration

Serum NO was determined by the Griess reaction according to Rajaraman et al.^20^*.* Briefly, 100µLfromeachsamplewastransferredintoflat-bottomed96-wellenzyme-linked immunosorbentassay(ELISA) plate and 100 µL of Griess reagent (0.2% naphthylethylenediamine dihydrochloride (NEDD), and 2% sulphanilamide in 5% phosphoric acid) were added. The mixture was incubated at 21 °C for 10 min. Absorbance of the samples and standards was measured at 570 nm using ELISA reader (Dynatech MR7000, Dynatech Laboratories Inc., Billinghurst, UK). The absorbance of test samples was converted to micromolar of nitrite by comparison with absorbance values of sodium nitrite standard curve within a linear curve fit.

#### Detection of serum cytokines

The serum cytokines; tumor necrosis factor-alpha (TNF-α), interferon gamma (IFN-γ) and interleukin-1 beta (IL-1β), were measured by specific ELISA kits (Elabscience, USA catalog numbers E-EL-R2856, E-EL-R0009, and E-EL-R0012, respectively) based on quantitative sandwich technique. The optical density values were read in a micro plate reader at 450 nm. The concentrations of cytokines in samples were determined by comparing the OD of the samples to the standard curve of each measured cytokine. The results were expressed as pg/mg.

#### Histopathological examination

Histopathological assessment was performed on 2–3 spleen and thymus tissues of rats randomly selected from each group. Organs were removed, washed with ice-cold saline, and placed in 10% formalin in saline. Tap water was used for washing, and then serial dilutions of alcohol were prepared for dehydration. The specimens were cleared in xylene then embedded in paraffin at 56 °C in a hot air oven for 24 h. Organs were kept until they become hard enough to be sectioned. Sections of 5 µm were cut by a sledge microtome, gathered on glass slides, then deparaffinized and stained with hematoxylin and eosin (H & E) for standard histological examination utilizing the light electric microscope according to the method of Hook et al.^21^.

### Statistical analysis

All the results were represented as mean ± standard deviation. Graph Pad Prism for Windows (version 6.0, Graph Pad Software, Inc., San Diego, CA) was used for the statistical analysis. One-way analysis of variance (ANOVA) followed by Tukey *post-hoc* test was used to determine the significance of difference between the studied groups. *P* ≤ 0.05 was considered statistically significant.

### Ethics approval and consent to participate

This study was approved by ethical committee of Faculty of Pharmacy, Ain Shams University, Egypt (Protocol approval number: ACUC-FP-ASU-RHDIRB2020110301- Nr. 38). and have therefore been performed in accordance with the ethical standards laid down in compliance with relevant guidelines the revised Animals (Scientific Procedures) Act 1986 in the UK and Directive 2010/63/EU in Europe).

## Results

### Hematologic parameters

Results of the hematological parameters in the various experimental groups treated with the five mushroom extracts each with low dose (200 mg/kg) and high dose (400 mg/kg) in comparison to the control groups are shown in Table 1. The five mushroom extracts showed a marked increase in the WBC counts and the lymphocytic counts compared to the control group. On the other hand, the hemoglobin (Hb) concentration showed a slight increase after intake of the mushroom species compared to the control group.

**Table 1:** Hematologic parameters in rats administered the mushroom extracts as compared to controls.

Group		WBC (10^9^/L)	LYM (%)	RBC (10^12^/L)	HGB (g/dL)	HCT (%)	PLT (10^9^/L)
1	Normal control (NaCl 0.9%)	4.15 ± 0.41	70.57 ± 1.65	6.67 ± 0.20	13.6 ± 0.08	36.48 ± 0.29	684.1 ± 62.16
2	Dexamethasone (0.1 mg/Kg)	2.7 ± 0.60*^@^	45.27 ± 2.78*^@^	7.48 ± 0.69*^@^	15.55 ± 2.09	39.55 ± 5.56	751.5 ± 213.60*
3	*Echinacea* (30 mg/Kg)	5.06 ± 2.23^#^	75.95 ± 10.4^#^	6.61 ± 0.39^#^	13.58 ± 0.88	36.09 ± 2.86	779.6 ± 36.77*^#^
4	*P. ostreatus* (200 mg/Kg)	6.43 ± 1.74*^#@^	69.45 ± 12.09^#@^	6.09 ± 0.55^#^	11.33 ± 0.82^#^	31.28 ± 2.44^#^	821.5 ± 128.32.*^#@^
5	*P. ostreatus* (400 mg/Kg)	7.8 ± 1.10*^#@^	77.2 ± 6.77*	6.88 ± 0.27	13.13 ± 1.33	34.5 ± 3.33^#^	710.3 ± 55.72*^#@^
6	*P. columbinus* (200 mg/Kg)	4.75 ± 1.678^#@^	70.9 ± 8.94^#@^	6.62 ± 0.44	13.48 ± 0.87	35.43 ± 2.07	824.8 ± 63.84*^#@^
7	*P. columbinus* (400 mg/Kg)	6.75 ± 2.63*^#@^	74.76 ± 3.33*^#^	6.50 ± 0.30	12.51 ± 0.86	32.8 ± 2.1^#^	891 ± 132.62*^#@^
8	*P. sajor-caju* (200 mg/Kg)	4.5 ± 0.97^#@^	76.35 ± 17.38*^#^	6.25 ± 0.76^#^	12.01 ± 0.59	32.16 ± 1.70^#^	762.5 ± 175.421*^#@^
9	*P. sajor-caju *(400 mg/Kg)	6.46 ± 1.14*^#@^	79.16 ± 7.63*^#^	6.63 ± 0.51	12.96 ± 0.90	34.05 ± 2.17^#^	779.1 ± 156.465*^#^
10	*L. edodes *(200 mg/Kg)	5.58 ± 2.77*^#^	69.25 ± 10.41^#@^	7.22 ± 0.76	14.16 ± 1.08	35.33 ± 3.125	905.5 ± 213.40*^#@^
11	*L. edodes *(400 mg/Kg)	7.35 ± 0.92*^#@^	75.33 ± 19.48*^#^	6.95 ± 0.56	13.81 ± 1.09	35.38 ± 2.10	811.1 ± 210.467*^#@^
12	*A. bisporus* (200 mg/Kg)	4.63 ± 0.85^#@^	67.14 ± 5.61*^#@^	6.51 ± 0.25	13.51 ± 0.98	34.48 ± 2.21^#^	816.8 ± 198.51*^#@^
13	*A. bisporus* (400 mg/Kg)	6.46 ± 0.23*^#@^	73.96 ± 4.11*^#^	6.56 ± 0.57	12.61 ± 0.39	32.01 ± 0.44^#^	891.6 ± 158.76*^#@^

Data are mean ± SD (n = 6). *HCT* haematocrit, *Hb* haemoglobin, *PLT* platelet, *RBC* red blood cell, *WBC* white blood cell, *LYM* Lymphocytes. ^*^Significantly different from normal control group, ^#^significantly different from dexamethasone control group, ^@^significantly different from Echinacea extract treated group at *P* ≤ 0.05.

### Immunomodulatory biomarker assay

Measurement of non-specific immune response parameters was done by evaluation of the lysozyme, NO, TNF-α, IFN-γ and IL-1 β concentrations in rat serum. Results indicated that immunomodulatory biomarker levels increased, variable degrees, in the groups force-fed on mushroom extracts with different doses (Table 2). High dose (400 mg/kg) of *P. ostreatus* has shown a significant immunostimulant effect indicated a 31% increase in lysozyme activity as compared to the control group and an increase 13% higher than that induced by group receiving *Echinacea* extract. Similarly, *P. sajor-caju* high dose, demonstrated an increase in the lysozyme activity by 21% relative to the control group 1 that received only normal saline and by 5% when compared to *Echinacea* extract. Quantification of the serum NO concentrations indicated that oral administration of the mushroom extracts increased NO levels at variable levels (Table 2). *P. sajor-caju* doubled NO production when compared to the dexamethasone only control group. *P. columbinus* also showed an increased immune response compared to dexamethasone control group and to the *Echinacea* extract control group.

**Table 2:** Immunomodulatory serum biomarkers of the tested mushroom extracts as compared to controls.

Group		LYS (µg/mL)	NO (µg/mL)	TNF-α (pg/mL)	IFN-γ (pg/mL)	IL-1β (pg/mL)
1	Normal control (NaCl 0.9%)	213.15 ± 2.84	4.31 ± 0.2	165.92 ± 5.1	78.62 ± 7.3	170.85 ± 10.04
2	Dexamthasone (0.1 mg/Kg)	184.29 ± 15.67*^@^	2.72 ± 0.06*^@^	145.32 ± 7.6*^@^	58.49 ± 6.9*^@^	121.7 ± 1.32*^@^
3	*Echinacea* (30 mg/Kg)	256.54 ± 20.31*^#^	4.11 ± 0.3^#^	237.2 ± 6.7*^#^	96.002 ± 7.1*^#^	222.39 ± 5.1*^#^
4	*P. ostreatus* (200 mg/Kg)	261.19 ± 11.30*^#^	5.24 ± 0.22*^#@^	180.51 ± 10.82*^#@^	79.55 ± 6.5^#@^	143.92 ± 10.5*^#@^
5	*P. ostreatus* (400 mg/Kg)	201.97 ± 12.09^#@^	5.79 ± 0.29*^#@^	226.08 ± 5.2*^#^	101.86 ± 5.9*^#@^	167.175 ± 6.8^#@^
6	*P.columbinus* (200 mg/Kg)	222.49 ± 14.09^#^	4.515 ± 0.25*^#^	189.591 ± 11.3*^#@^	68.93 ± 6.5*^#@^	145.24 ± 2.55*^#@^
7	*P. columbinus* (400 mg/Kg)	239.06 ± 14.05*^#^	6.15 ± 0.12*^#@^	203.96 ± 4.4*^#@^	120.29 ± 5.4*^#@^	160.08 ± 3.79*^#@^
8	*P.sajor-caju* (200 mg/Kg)	259.05 ± 12.02*^#^	5.3 ± 0.2*^#@^	168.41 ± 8.4^#@^	56.11 ± 4.6*^@^	142.79 ± 7.3*^#@^
9	*P. sajor-caju* (400 mg/Kg)	250.75 ± 16.03*^#^	6.30 ± 0.35*^#@^	185.91 ± 5.4*^#@^	92.85 ± 2.3*^#^	209.71 ± 9.04*^#^
10	*L. edodes* (200 mg/Kg)	238.4 ± 13.95*^#^	4.16 ± 0.04^#^	122.82 ± 10.23*^#@^	60.68 ± 0.21*^#@^	150.54 ± 3.32*^#@^
11	*L. edodes* (400 mg/Kg)	204.14 ± 11.9^#@^	4.32 ± 0.16^#^	169.68 ± 11.92^#@^	74.48 ± 1.20^#@^	130.17 ± 8.18*^#@^
12	*A. bisporus* (200 mg/Kg)	250.12 ± 12.63*^#^	3.39 ± 0.15*^#^	149.75 ± 10.28*^@^	75.09 ± 7.47^#@^	103.77 ± 3.65*^#@^
13	*A. bisporu*s (400 mg/Kg)	245.48 ± 19.75*^#^	5.56 ± 0.29*^#@^	170.92 ± 11.29^#@^	61.02 ± 1.08*^#@^	114.58 ± 2.49*^@^

Data are mean ± SD (n = 6). *LYS* lysozyme, *NO* Nitric oxide, *TNF-α* tumor necrosis factor alpha, *IFN-γ* Interferon gamma, *IL-1β* interleukin 1 beta. ^*^Significantly different from normal control group, ^#^significantly different from dexamethasone control group, ^@^significantly different from *Echinacea* extract treated group at *P* ≤ 0.05.

As displayed in Table 2, the levels of TNF-α in the serum of the tested rats demonstrated a significant increase after administration of the high dose of *P. ostreatus* extract. This increase was by 56% and 37% compared to the dexamethasone only fed control group and untreated control group, respectively. However, no significant difference was demonstrated from the *Echinacea* extract control group. Moreover, *P. columbinus* showed an increase by 22% from the control group and by 31% from the dexamethasone only fed group but still less than group immunostimulated with *Echinacea* extract.

The IFN-γ concentration in the blood serum of the tested rats revealed a significant increase post administration of the different mushroom extracts. *P. columbinus* showed an increase by 53% from the control group and a marked increase by 90% from the dexamethasone only fed group. Another marked increase in IFN-γ concentration was seen in rats fed with *P. ostreatus* extrac*t* with 74% from the dexamethasone control group, but no significant difference was demonstrated from the *Echinacea* extract control group*.* On the other hand, *P. sajor-caju* at low dose group showed a decrease in IFN-γ concentration just like the dexamethasone control group.

Finally, the IL-1β concentration levels in rat serum revealed variable immunomodulatory characters after the rats were fed on the different mushroom extracts as shown in Table 2. *P. sajor-caju* high dose group showed increase from the normal control group by 22% and by 72% marked increase from the dexamethasone control group. In contrast*, A. bisporus* fed groups showed a decrease in the IL-1β serum concentration compared to normal and dexamethasone control groups.

### Histopathological findings

**Figure 1:**
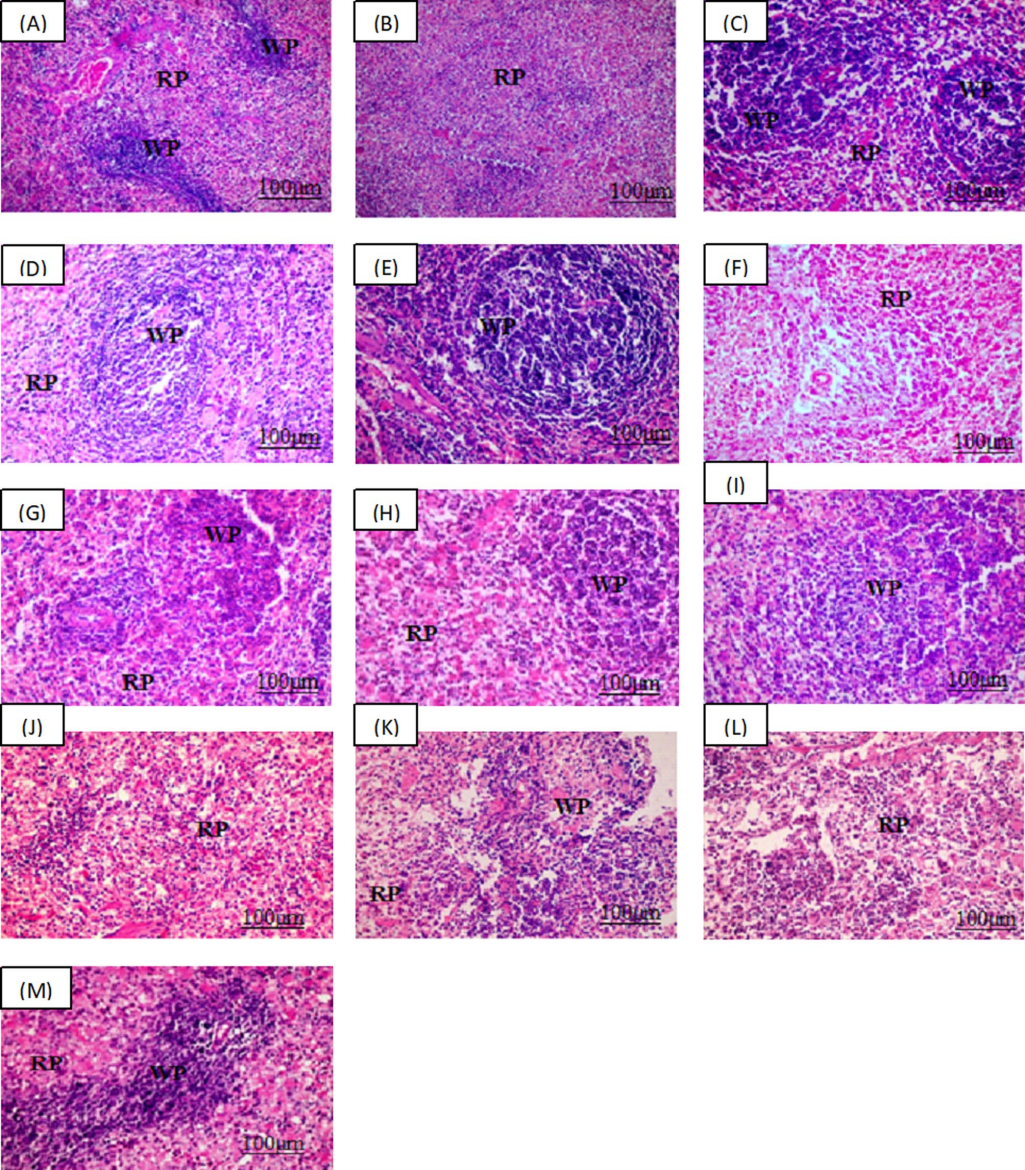
Histopathological Findings of the spleen. Group 1 (**A**) for normal control group showing normal white pulp (WP) and red pulp (RP), Group 2 (**B**) for dexamethasone control group. Group 3 (**C**) for *Echinacea* extract treated group. *Pleurotus ostreatus* group for low dose (200 mg/Kg) Group 4 (**D**) and high dose (400 mg/Kg) Group 5 (**E**). *Pleurotus columbinus* low dose (200 mg/Kg) Group 6 (**F**) and high dose (400 mg/Kg) Group 7 (**G**). *Pleurotus sajor-caju* for low dose (200 mg/Kg) Group 8 (**H**) and high dose (400 mg/Kg) Group 9 (**I**). *Lentinula edodes* low dose (200 mg/Kg) Group 10 (**J**) and high dose (400 mg/Kg) Group 11 (**K**). *Agaricus bisporus* low dose (200 mg/Kg) Group 12 (**L**) and high dose (400 mg/Kg) Group 13 (**M**) (H& E).

**Figure 2:**
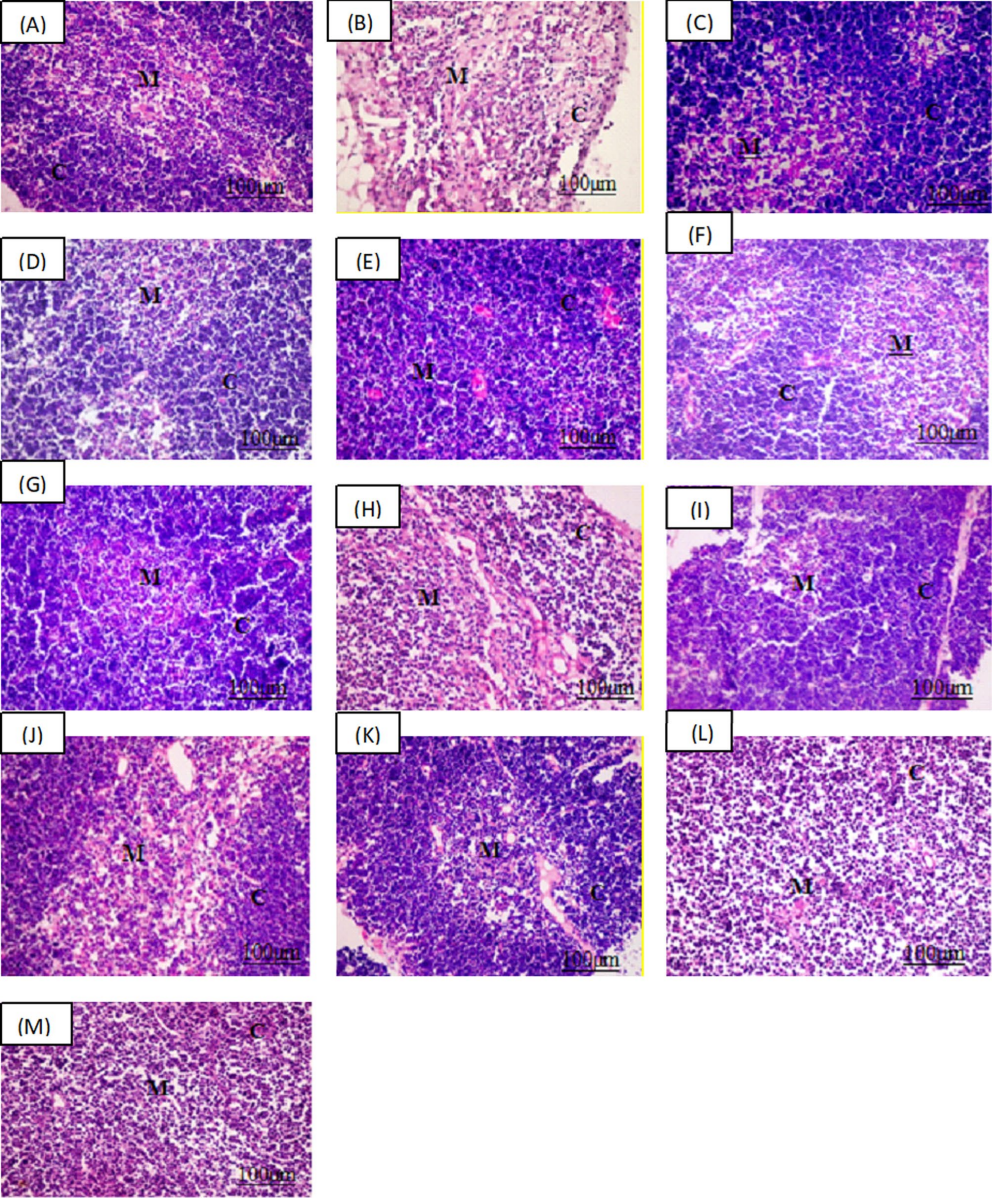
Histopathological findings of the thymus. Group 1 (**A**) for normal control group showed normal cortex (**C**) and medulla (M), Group 2 (**B**) for dexamethasone control group. Group 3 (**C**) for *Echinacea* extract treated group. *Pleurotus ostreatus* group for low dose (200 mg/Kg) Group 4 (**D**) and high dose (400 mg/Kg) Group 5 (**E**). *Pleurotus columbinu*s low dose (200 mg/Kg) Group 6 (**F**) and high dose (400 mg/Kg) Group 7 (**G**). *Pleurotus sajor-caju* for low dose (200 mg/Kg) Group 8 (**H**) and high dose (400 mg/Kg) Group 9 (**I**). *Lentinula edodes* low dose (200 mg/Kg) Group 10 (**J**) and high dose Group (400 mg/Kg) 11 (**K**). *Agaricus bisporus* low dose (200 mg/Kg) Group 12 (**L**) and high dose (400 mg/Kg) Group 13 (**M**). (H&E).

Group 1 showed normal histopathological features in both spleen and thymus. Spleen showed the control group white pulp of rats was characterized by clearly differentiated T cell areas that surround the central arteries, forming periarteriolar lymphoid sheath (PALS). Besides, the follicles contain germinal centers with present mitoses. There was a mantle zone at the follicle periphery beyond which there was an immense marginal zone. The marginal sinus was also clearly discriminated and the white and the red pulp in between boundary were well expressed in Fig. 1A. Normal thymus in normal control group in Fig. 2B is formed of darkly stained cortex contains heavy packed, small, and immature lymphocytes, which overshadow the population of the sparse epithelial cell. Paler staining less densely cellular content was seen in the medulla, than the cortex, and contains more mature lymphocytes, prominent epithelial cells, admixed macrophages, Hassall’s corpuscles, and dendritic cells.

While in group 2, spleen showed absence of complete germinal centers in white pulp in spleen tissue of all samples, they were represented rarely and had smaller dimensions, with lymphocyte depletion. Trabeculae smooth muscle cells diminution and collagen fibers dominance was notable in all experimental groups when compared with the control group. Dexamethasone caused pronounced decrease of the volume density of the lymph follicles and a significant increase in the red pulp in Fig. 1B. Thymus showed decrease in cortical lymphocytes cellular density and deteriorating changes in the remaining epithelial and stromal cells are displayed by cytoplasmic vacuolization in dexamethasone control group in Fig. 2B.

In group 3, the spleen showed apparent normal splenic white pulp with the central arteries surrounded by clearly differentiated T cell areas, forming periarteriolar lymphoid sheath (PALS), with slight depletion of its lymphocytes. Many follicles possess germinal centers and the borderline between the white and the red pulp was well expressed in *Echinacea* extract treated group in Fig. 1C. Thymus in Fig. 2C showed apparent normal structure with densely populated developing T cells in thymic cortex as well as a smaller proportion of associated epithelial cells. Mature and larger T cells were frequently existed in the medulla where epithelial and other cell types were more abundant in *Echinacea* extract treated group.

In groups 4, 6, 8, 10, 12 at which rats were administered the mushroom extracts in low doses of 200 mg/kg, spleens showed moderate white pulp depletion with increase the number of reticuloendothelial cells in red pulp and thymus showed mild reduced in lymphocytes population with mild lymphocytes apoptosis and macrophages phagocytized apoptotic debris as seen in Figs. 1 and 2D, F, H, J and I of *Pleurotus ostreatus, Pleurotus columbinus, Pleurotus sajor-caju, Lentinula edodes* and *Agaricus bisporus* respectively. On the other hand, groups 5, 7, 9, 11, 13 where mushroom was fed to rats in high doses of 400 mg/kg, spleen showed apparent normal white pulp with absence of germinal centers, increase number of lymphoblast, and lymphocytes were arranged in clusters. Some lymphocytes undergo apoptosis with proliferation of reticuloendothelial cells especially macrophages in red pulp and thymus showed moderate increase in lymphocytes population in both cortex and medulla with macrophages phagocytized apoptotic debris shown in Figs. 1 and 2E, G, I, K and M of *P. ostreatus, P. columbinus, P. sajor-caju, L. edodes* and *A. bisporus,* respectively.

## Discussion

Macrofungi, mostly from their fruiting bodies which are generally referred to as mushrooms, are commonly cultivated all over the world and are widely used as food. The most cultivated edible mushrooms globally are *A. bisporus*, *L. edodes*, *Flammulina velutipes*, and *Pleurotus* spp.^22^. As a consequence of their richness in many nutrients, mushrooms have been associated with activating the immune system^23^. Various biological active substances have been found in mushroom fruits or mycelia, such as carotenoids, alkaloids, lectins, enzymes, folates, fats, organic acids, minerals, polysaccharides, phenolics, proteins, tocopherols, terpenoids, and volatile compounds in general^24,25^.

Natural immunotherapies are considered encouraging alternatives to chemotherapy and vaccines due to their wide spectrum activity, eco-friendly measures, and cost effectiveness. In the present study, the effects of the mushroom extracts on potentiation of the immune system were investigated after 15 days of successive dietary supplementation in Wistar rat albino.

The immune system major components are monocytes, granulocytes, macrophages, and humoral elements, such as immunoglobulin, the complement system and lysozymes^26^. In the cellular immunity and infectious diseases resistance, white blood cells (WBCs) play a critical role. Functions of leukocyte can be enhanced by natural immunostimulants as was previously reported^27^. In the current study, we determined the immune components and mediators in Wistar rats fed in our challenged mushroom extracts by immunosuppressant drug, dexamethasone. Our results showed that there was a significant change in the hematologic parameters after Wistar rats were force-fed on the different mushroom extracts. The WBCs were significantly increased at high doses of 400 mg/kg of the five mushroom extracts. *P. ostreatus* and *L. edodes* showed a marked increase in the WBCs compared to the untreated and negative control groups with moderate increase from the positive control group. Lymphocytic percentage also showed a slight increase in the Wistar rat groups force-fed on the mushroom species compared to the untreated group. *P. sajor-caju* 400 mg/kg dose showed a raise in lymphocytic percentage even when compared to positive control group (79% compared to 76% for *Echinacea* control group).

In accordance with our findings, leucopoiesis enhancement potential showed in a previous study by Ajith et al. showed that metabolites from *P. ostreatus* and *P. pulmonarius* had the ability to raise the total count of leukocyte in Wister rats, where the group that received the highest dose (80 mg/mL) showed the highest leukocyte count^28^. The results were corroborated by another study by Taveira et al. showing that extracts from the same mushrooms had profound antitumor activities as the polysaccharide complexes of mushrooms were capable of modulating the immune system and exerted antitumor activities^29^. Maity et al. also reported that the mechanism of action of mushroom polysaccharides was to stimulate T-cells, B-cells, natural killer cells, and macrophage dependent immune responses via binding to receptors like the toll-like receptor-2 and dectin-1^30^. In addition, several reports suggested that lentinan and β-glucan stimulates the proliferation of lymphocytes, monocytes and macrophages, where it has been demonstrated that receptors for β-glucans are expressed on neutrophils^31^. Other studies observed that the mushroom extracts activate the NK cells via the upregulation of IFN-γ and perforin production and increasing the expression of the activating receptor NKp30^32^.

Moreover, the current study used the lysozyme activity and the NO concentration assay as an evaluation to the immune response after exposure to the mushroom aqueous extracts. *P. ostreatus and P. sajor-caju* fed groups have shown a significant increase in the lysozyme activity compared to the normal control group and the dexamethasone fed group. Ragland et al. reported that polysaccahrides isolated from *Pleurotus* spp. Induced significantly higher levels of lysozyme release and NO production^33^. Various peptides, such as antibodies, lysozyme, complement factors, and other lytic factors, are present in serum as a first line of defense, preventing microbe adherence and colonization, therefore preventing infections and illnesses^34^. Our findings regarding NO production in the serum of tested rats showed that *P. sajor-caju* and *P. columbinus* had increased the serum NO concentrations after administration for 15 days. Other studies reported that, NO is generated in excess during the first step in macrophage response to the invading microorganism, as a result of host response against infections and inflammatory conditions^35^.

The present study, reported that the rat groups fed on *P. ostreatus and P. columbinus*, had increased the TNF-α, IFN-γ and IL-1β from the negative control group. Also, *P. sajor-caju* species demonstrated increase in IL-1β, IFN-γ and no significant increase in the TNF-α. As a member of interleukin-1 (IL-1) cytokine family, Interleukin-1β (IL-1β), is a prototypical pro-inflammatory cytokine and key mediator of the body’s reaction to microbial infection, immunological response and tissue damage. TNF-α, a pro-inflammatory cytokine, is one of the early immune genes expressed at the beginning of infection and has a key part in controlling inflammation^36^. Studies reported that, TNF-α shows overlapping functions with IL-1β.

TNF-αs have been generated in site of infection as monomers, dimers and trimmers and can enact macrophages/phagocytes and improve their killing activity against microorganisms^37^. Contrastingly, *L. edodes* and *A. bisporus* induced a minor increase in the level of serum cytokines. Mushroom bioactive compounds follow vast mechanisms to inflect immune system in cancer therapy. It was previously reported that aqueous extracts of *A. blazei* Murill (AbM) fruiting bodies stimulate synthesis of various cytokines^38^. Moreover, there was a prominent increase in granulocytes and monocytes leading to release of important interleukines after administration of Cauliflower mushroom confirming its immunomodulatory properties^38^.

Polysaccharides *from P. tuber-regium* and *P. rhinoceros* mushrooms were reported to have anticancer effects, due to their ability to increase the expression and proliferation of macrophages, NK cells, and T helper cells in mice^39^. Additionally, *G. lucidum*, a tonic plant, promotes the inflammatory response by expression and production of several chemokines^22,40,41^. Previous studies by our research team, have shown that the 5 tested mushroom extracts were rich in polysaccharides, vitamin C, flavonoids and phenolic compounds which have been linked to stimulation of the immune system^15,16^. Lentinan, a β-d-glucan from *L. edodes* mushrooms, improves the formation of Th1 immune responses through activating DCs maturation^42^. Temporarily, Tregs activation that results in Th1-mediated immune responses suppression is prevented by lentinan, which activates production of IL-10 and causes CD4 + T cells apoptotic reduction resulting in Th1 immune responses potentiation^43^. Besides, stimulation of host DCs causes T cell activation, resulting in the generation of adaptive immunological responses. Activation of DCs results in the production of IL-12, which aids in Th1 differentiation^44^. CD4 + T lymphocytes can stimulate macrophages to create large levels of NO by producing IFN-γ^45^. These findings point out that the DCs effective stimulation is crucial for developing Th1 protective immune response^46^. The histopathological findings have shown the signs of immune suppression in the spleen of dexamethasone control group expressed as absence of germinal centers in white pulp with lymphocyte depletion, trabecular smooth muscle cell reduction, and domination of collagen fibers. Thymus as well, showed decrease in cortical lymphocytes cellular density and regressive changes in the remaining epithelial and stromal cells are defined by vacuolization of the cytoplasm. It is worth mentioning that high doses of the five mushroom extracts showed similar effect to that of *Echinacea* extract in splenic and thymus tissues. Other studies observed that β-glucans and lentinan from *Hordeum vulgare, G. frondosa*, *Laminaria angustata*, *Lentinula edodes*, and α- glucans from *Pleurotus ostreatus* and *Sclerotinia sclerotiorum* improved the functions of the immune system in animal models in the bone marrow, spleen, gut, thymus, blood, liver, lungs, and saliva, moreover, controlled human studies recorded indication of immune stimulation in the blood^47–49^.

## Conclusion

This study demonstrated the in vivo immunostimulatory capacities of the aqueous extracts of five edible mushrooms including, *Lentinula edodes*, *Agaricus bisporus*, *Pleurotus ostreatus*, *Pleurotus columbinus*, and *Pleurotus sajor-caju* using Wistar albino rats. The resulted immunostimulatory activities were comparable to that obtained by the positive control, *Echinacea* extract as determined by assessment of various immunologic parameters including, lysozyme activity, NO production and serum cytokines (TNF-α, IFN-γ and IL-1β) levels. The immunostimulatory effects were also confirmed by histopathological examination of the rats' spleen and thymus tissues where marked lymphocytic proliferation was obvious particularly in *Pleurotus ostreatus, Agaricus bisporus* and *Lentinula edodes* at the higher doses (400 mg/Kg). The obtained preclinical findings should be confirmed clinically for potential therapeutic use in humans.

## Data Availability

All data are included within the manuscript.

## Abbreviations

ADCO: Arab drug company
BRMs: Biological response regulators
DCs: Dendritic cells
ELISA: Enzyme-linked immunosorbent assay
EDTA: Ethylenediaminetetraacetic acid
HCT: Hematocrit
Hb: Hemoglobin
IL: Interleukin
IL-1β: Interleukin-1 beta
IFN-γ: Interferon gamma
ITS: Internal transcribed spacer
LYM: Lymphocytes
LYS: Lysozyme
NEDD: Naphthylethylenediamine dihydrochloride
NO: Nitric oxide
PLT: Platelet
RBC: Red blood cell
RP: Red pulp
TNF-α: Tumor necrosis factor-alpha
WBC: White blood cell
WP: Normal white pulp
